# Special Issue: Challenges and Future Trends of COVID-19 Vaccination

**DOI:** 10.3390/vaccines12121425

**Published:** 2024-12-18

**Authors:** Imran Mirza

**Affiliations:** United Nations Children’s Fund (UNICEF) HQ, New York, NY 10017, USA; imirza@unicef.org

During the COVID-19 pandemic, over 13.5 billion vaccine doses were administered globally in record time. This unprecedented effort required innovative approaches to reduce morbidity and mortality, safeguard health systems, and quickly restore socio-economic activities, but it caused an additional burden on strained public health systems across the world, with service disruptions observed across the range of primary health care (PHC) and immunization services.

The global rollout of COVID-19 vaccines revealed the existing complexities and vulnerabilities of immunization systems, including addressing challenges like vaccine hesitancy, misinformation, socio-cultural barriers, missed communities, and integration with routine healthcare systems. Emerging themes from recent studies highlight the importance of generating, analyzing, and using behavioral and social data to inform community-based strategies, policy initiatives, and culturally sensitive communication to improve vaccine acceptance.

Vaccine acceptance is contextual and influenced by misinformation, cultural beliefs, and historical mistrust of healthcare systems. Studies show that hesitancy varies significantly across demographic, socio-economic, and regional lines [1]. Trust in health authorities and knowledge about vaccines are key enablers of vaccination willingness, underscoring the importance of health literacy and governmental credibility. For instance, in Yemen, vaccine acceptance was linked to socio-political instability and limited healthcare resources [2]. In other regions, such as Italy, vaccine safety concerns and historical public health policy experiences shaped attitudes, emphasizing the need for transparent and culturally informed communication strategies [3,4].

Targeted communication, grounded in empathy and cultural relevance, is essential. Community-based approaches—such as bringing vaccinators into trusted local environments—have shown promise in fostering vaccine acceptance. Studies have also demonstrated the effectiveness of community-based digital health interventions in countering misinformation and encouraging vaccination [1,5]. In marginalized areas, empowering community health workers with resources and training is crucial for navigating sensitive conversations and building trust. Additionally, engaging local influencers, religious leaders, and grassroots organizations has been instrumental in reshaping community attitudes toward immunization. Insights from diverse contexts further emphasize the need for locally tailored strategies. For example, hesitancy among healthcare workers in Somalia was found to be influenced by professional roles, education levels, and prior exposure to COVID-19 [6]. Similarly, U.S.-based studies [7] revealed concerns about vaccinating younger populations due to safety fears, mirroring also global challenges in child immunization.

To address these concerns related to vaccine hesitancy and increase acceptance, policymakers should consider establishing clear frameworks for multi-stakeholder collaborations that include community leaders, local influencers, and healthcare providers. Strengthening these collaborations can help sustain demand for COVID-19 vaccines and other immunizations, especially in the face of fluctuating public interest as the pandemic stabilizes, along with empowering healthcare providers with accurate, tailored, empathetic communication and developing long-term strategies to shift attitudes over time.

The integration of COVID-19 vaccination into PHC systems is critical for sustainability, particularly as the pandemic has now transitioned to an endemic phase; hence, vaccination efforts have propelled broader discussions on integrating such immunizations into routine health services and has encouraged policymakers to reconsider how vaccines are integrated into primary health care (PHC) systems. However, evidence of effective integration remains sparse, particularly for vulnerable groups such as pregnant women, elderly, and individuals with co-morbidities. Large-scale campaigns combined with routine immunization services have been effective in boosting coverage in Africa, though these efforts require not only logistical resources but also significant investments in health worker training, local community engagement, data generation, analysis and management, and sustainable funding sources [8].

Future immunization policies must prioritize sustainable platforms over short-term campaigns. Aligning immunization efforts with broader health system strengthening initiatives, as exemplified by a study, can create more resilient healthcare systems with far-reaching benefits [9]. Integrating immunization with routine services, such as childhood health screenings, can reduce logistical barriers, improve accessibility, and position vaccination as a routine part of healthcare delivery. To support this, policies must ensure adequate funding, cold-chain infrastructure, and community engagement while promoting real-time data sharing and transparency for targeted interventions [8,9].

The global experience with COVID-19 vaccination highlights the critical need for adaptive, community-based strategies that respect diverse geographical and socio-cultural contexts. Building trust in vaccines and addressing barriers to vaccine uptake requires more than disseminating information; it demands partnerships with trusted local figures, culturally aligned messaging, and policies that support integrated health systems. Challenges like safety concerns, misinformation, and structural barriers must be met with resilient and responsive approaches, but most importantly high-quality services including vaccines should be made available to populations.

By incorporating these lessons, using existing public health delivery platforms and community social structures, public health systems can strengthen immunization infrastructure, enhance vaccine equity, and lay the groundwork for robust pandemic responses and resilient routine immunization programs in the future. In addition, the delivery of COVID-19 vaccines has created an opportunity to enhance adult vaccination strategies and integrate them with other healthcare programs, fostering holistic immunization services across the life course, ultimately contributing to improved health outcomes, sustained population immunity, and ensures that no one is left behind in the global fight against COVID-19, other infectious, and non-communicable diseases.
